# Nitrate Reductase Genes *AtNIA1* and *AtNIA2* Confer Heat Stress Resilience via ROS Homeostasis and HSP Expression in *Arabidopsis*

**DOI:** 10.3390/biom16030415

**Published:** 2026-03-11

**Authors:** Nusrat Jahan Methela, Mohammad Shafiqul Islam, Mahir Faysal, Moon-Sub Lee, Byung-Wook Yun, Bong-Gyu Mun

**Affiliations:** 1 Department of Agriculture, Noakhali Science and Technology University, Noakhali 3814, Bangladesh; methela.ag@nstu.edu.bd (N.J.M.); shafik.ag@nstu.edu.bd (M.S.I.); 2 Department of Computer Science & Engineering, Daffodil International University, Dhaka 1216, Bangladesh; 3 Department of Crop Science, Chungbuk National University, Cheongju 28644, Republic of Korea; 4 Department of Applied Biosciences, College of Agriculture and Life Sciences, Kyungpook National University, Daegu 41566, Republic of Korea; 5 Department of Environmental and Biological Chemistry, Chungbuk National University, Cheongju 28644, Republic of Korea

**Keywords:** *Arabidopsis thaliana*, heat stress, *Arabidopsis* mutant, nitric oxide, nitrate reductase, oxidative stress

## Abstract

Heat stress is a key environmental factor that adversely affects plant growth, development, and productivity. Nitrate reductase (NR), encoded by *AtNIA1* and *AtNIA2*, plays a crucial role in nitric oxide (NO) biosynthesis, which mediates stress responses in plants. In this study, we investigated the roles of AtNIA1 and AtNIA2 in regulating plant heat stress tolerance. Under heat stress conditions, *Arabidopsis thaliana* plants maintained higher relative water content and chlorophyll levels, whereas atnia1 and atnia2 mutants exhibited greater physiological damage. Oxidative stress markers such as MDA and H_2_O_2_ accumulated to higher levels in nitrate reductase mutants than in Col-0, indicating increased heat sensitivity. Gene expression analysis further revealed a pronounced late-phase induction of MBF1c in atnia2 plants, accompanied by altered expression of heat shock proteins. These results suggest that nitrate reductase-dependent pathways contribute to heat stress tolerance by regulating water status, membrane stability, ROS detoxification, and heat shock gene expression. This study provides new insights into NR-mediated NO signaling in thermotolerance and highlights potential targets for improving crop resilience under rising temperatures.

## 1. Introduction

Escalating global temperatures due to climate change pose a significant threat to agricultural productivity and ecosystem stability [1]. Among the various abiotic stressors; heat stress is particularly detrimental to plant growth and development; affecting key physiological and biochemical processes such as photosynthesis; membrane stability; and metabolic homeostasis [2,3]. High temperature induces excessive production of reactive oxygen species (ROS), which can damage lipids, proteins, and nucleic acids, ultimately leading to cellular dysfunction and growth inhibition. As sessile organisms, plants have evolved intricate defense systems involving antioxidants, stress-responsive genes, and signaling molecules to mitigate heat-induced damage [4]. However, the underlying molecular components and regulatory pathways that govern heat stress tolerance are still being unraveled. Nitrate reductase-dependent nitric oxide production has been implicated in multiple abiotic stress responses; including oxidative stress and temperature stress. Given the established role of NIA1 and NIA2 in NO homeostasis; investigating their contribution to heat stress tolerance provides an opportunity to better understand the transcriptional and physiological mechanisms underlying stress adaptation and thermotolerance.

One critical signaling molecule in plant abiotic stress responses is nitric oxide (NO), a gaseous free radical with multifaceted roles in development, defense, and redox homeostasis [5,6]. Under stress conditions, NO interacts with ROS to modulate antioxidant enzyme activities, regulate ion channels, and trigger transcriptional reprogramming of protective genes [7,8]. In *Arabidopsis thaliana*, one of the primary enzymatic sources of NO is nitrate reductase (NR), which catalyzes the reduction in nitrate to nitrite [9]. The NR family in Arabidopsis comprises two isoforms, *AtNIA1* and *AtNIA2*. While both genes are involved in nitrate assimilation, emerging evidence suggests functional divergence, where *AtNIA1* plays a more prominent role in stress-induced NO production, whereas *AtNIA2* contributes largely to nitrogen metabolism under normal physiological conditions [9,10]. Previous studies have demonstrated that *atnia1nia2* double mutants exhibit elevated oxidative damage, reduced antioxidant enzyme activities, and altered expression of stress-related genes under salinity, drought, and cold stress [11,12,13,14]. However, despite the growing interest in NO-mediated thermotolerance, the individual roles of *AtNIA1* and *AtNIA2* in heat stress response remain poorly understood. Given their differential regulation and distinct physiological roles, it is plausible that *AtNIA1* and *AtNIA2* may contribute unequally to plant thermoprotection via NO signaling.

In this study, we aimed to dissect the isoform-specific contributions of *AtNIA1* and *AtNIA2* to heat stress tolerance in *Arabidopsis thaliana*. Using wild-type Col-0 along with *atnia1* and *atnia2* knockout mutants, we systematically evaluated plant responses under acute heat stress by assessing oxidative stress markers (MDA, H_2_O_2_), chlorophyll content, and expression of heat-responsive and multibinding factor-related genes. Our findings reveal that *atnia1* mutants exhibit heightened sensitivity to heat stress compared to *atnia2*, as reflected by increased oxidative damage, reduced chlorophyll retention, and impaired transcriptional activation of stress-related genes. These results suggest that *AtNIA1* plays a dominant role in modulating thermotolerance, likely through NO-mediated redox regulation and stress gene activation. This study provides novel insights into the distinct physiological functions of nitrate reductase isoforms in plant heat stress adaptation and highlights *AtNIA1* as a potential target for improving thermotolerance in crops.

## 2. Materials and Methods

### 2.1. Plant Material and Growth Condition

Seeds of *Arabidopsis thaliana* ecotype Columbia (Col-0) and the loss-of-function mutants *AtNIA1* and *AtNIA2* were obtained from the Nottingham *Arabidopsis* Stock Centre (NASC). All lines used in this study were in the Col-0 genetic background. Seeds were sown in soil and grown for 28 days in a controlled growth chamber at 24 ± 1 °C under long-day conditions (16 h light/8 h dark) with white fluorescent tube lights (~100 µmol m^−2^ s^−1^) and 62 ± 2% relative humidity. For heat stress treatment, plants were exposed to 37 °C for 6 h during the day, followed by 24 °C for the remainder of the day, over a period of 5 consecutive days. Plant samples were then collected, immediately snap-frozen in liquid nitrogen, and stored at −80 °C for further analyses.

### 2.2. Relative Water Content, Total Chlorophyll and Electrolyte Leakage

Relative water content (RWC) was determined following the method described by [15,16] with minor modifications. Whole plant samples were collected and rinsed three times with distilled water to remove surface contaminants, after which the fresh weight (FW) was immediately recorded. The samples were then fully hydrated in distilled water for 2 h at room temperature, blotted dry, and weighed to obtain the turgid weight (TW). Subsequently, samples were oven-dried at 65 °C for at least 48 h until a constant dry weight (DW) was achieved. RWC was calculated using the equation:

RWC (%) = ((FW − DW)/(TW − DW)) × 100

Chlorophyll content was determined using a chlorophyll concentration meter (MC-100, Apogee Instruments Inc., Logan, UT, USA) and expressed as μmol of chlorophyll per m^2^ [6]. Electrolyte leakage (EL) was measured following the method of [5], with minor modifications. Two leaf discs (1 cm in diameter) were excised from each plant. Six discs from the same genotype were pooled to constitute one biological replicate. The discs were rinsed thoroughly with distilled water to remove surface-adhered electrolytes and placed in test tubes containing 10 mL of deionized water. Samples were incubated at 25 °C for 2 h, after which the initial electrical conductivity (L_1_) of the bathing solution was measured using a portable conductivity meter (HORIBA Twin Cond B-173, Kyoto, Japan). The samples were then autoclaved at 120 °C for 20 min to release all electrolytes, cooled to 25 °C, and the final conductivity (L_2_) was recorded. Electrolyte leakage was calculated as follows:

EL (%) = L1/L2 × 100

### 2.3. Measurement of MDA, CAT, H_2_O_2_, and APX Activity

For malondialdehyde (MDA) and hydrogen peroxide (H_2_O_2_) quantification, 250 mg of fresh leaf tissue was homogenized in 1.5 mL of 1% (*w*/*v*) trichloroacetic acid (TCA) and centrifuged at 10,000× *g* for 15 min at 4 °C. MDA determination was performed as described by [17,18]. The supernatant was mixed with reaction buffer (1:2, *v*/*v*) containing 20% TCA and 0.5% thiobarbituric acid (TBA), incubated at 95 °C for 1 h, rapidly cooled in an ice bath for 5 min, and centrifuged at 10,000× *g* for 10 min. Absorbance was recorded at 532 nm and corrected for nonspecific turbidity at 600 nm using a spectrophotometer (Shimadzu UV-1280, Kyoto, Japan). MDA concentration was calculated using an extinction coefficient of 155 mM^−1^ cm^−1^ and expressed as μmol MDA g^−1^ fresh weight (FW). H_2_O_2_ quantification was performed according to [19]. The reaction mixture (3 mL total volume) contained 500 μL of 100 mM potassium phosphate buffer, 500 μL of leaf extract supernatant, and 2 mL of 1 M potassium iodide (added last). Samples were incubated for 1 h in the dark, followed by 20 min at room temperature, and absorbance was measured at 390 nm. H_2_O_2_ concentration was determined using a standard curve.

Catalase (CAT) activity was assayed in a 1 mL reaction mixture comprising 100 mM sodium phosphate (Na_2_HPO_4_) buffer (pH 7.4), 10 mM H_2_O_2_, and 20 µg of protein extract from control and treated plant samples [20,21]. The decomposition of H_2_O_2_ was monitored spectrophotometrically at 240 nm, using an extinction coefficient of 39.4 mM^−1^ cm^−1^. Ascorbate peroxidase (APX) activity was assayed following [22,23] by monitoring the decrease in absorbance at 290 nm (extinction coefficient: 2.8 mM^−1^ cm^−1^). The reaction mixture (1 mL final volume) consisted of 20 μL of enzyme extract, 0.1 μM EDTA, 50 mM phosphate buffer, 0.5 mM ascorbate, and 1 mM H_2_O_2_.

### 2.4. Measurement of ABA and SNO

ABA content was measured following the procedure described by [24,25]. During sample preparation, an isotopically labeled ABA standard [(±)-3,5,5,7,7,7-d6] was added to enable accurate detection and peak comparison. Quantification was carried out using a 6890 N Gas Chromatograph coupled with mass spectrometry (GC-MS; Agilent, Santa Clara, CA, USA). Ion responses were analyzed using ThermoQuest software (Manchester, UK), monitoring *m*/*z* values of 162 and 190 for methylated ABA (Me-ABA) and 166 and 194 for the labeled methylated standard (Me-(^2^H_6_)-ABA). S-nitrosothiol (SNO) levels were measured using a Sievers NOA-280i analyzer, following the method described previously [26]. A total of 500 mg of tissue was homogenized in PBS and centrifuged at 750× *g* for 7 min. A 500 µL aliquot of the resulting supernatant was mixed with an equal volume of PBS in a fresh tube and then applied to Sephadex G-25 (NAP-25, Amersham, UK) columns by gravity flow to remove nitrate and low-molecular-weight thiols. S-nitrosothiols (SNOs) were subsequently quantified in 100 µL of the filtered eluate. Acidified KI buffer (5 mL acetic acid, 50 mg KI, 200 mM CuSO_4_) was employed in the purge vessel as a reducing agent during measurement.

### 2.5. RNA Extraction, cDNA Synthesis and Quantitative Real Time PCR

Total RNA was isolated from frozen Arabidopsis leaf samples using TRIzol reagent (MRC, Cincinnati, OH, USA) following the manufacturer’s protocol. cDNA was synthesized from 1 µg of total RNA using the SolGent DiaStar™ RT Kit (SolGent, Daejeon, Republic of Korea). Quantitative real-time PCR was conducted on a Bio-Rad CFX Duet Real-Time PCR System (Singapore) using Solg™ 2× Real-Time PCR Smart Mix with SYBR^®^ Green I [5]. PCR amplification conditions were initial polymerase activation at 95 °C for 15 min, followed by 40 cycles of 95 °C for 20 s (denaturation), 58–60 °C for 40 s (annealing, depending on primer Tm), and 72 °C for 30 s (extension). Relative transcript levels were calculated using *PP2A* (*AtPP2A*) as the internal reference gene [27,28]. Primer sequences are provided in Supplementary Table S1.

### 2.6. Statistical Analysis

All experiments were conducted with at least three biological replicates, each replicate comprises six plants. Data were presented as mean ± standard error (SE). For comparisons between two groups (e.g., control vs. heat within the same genotype), statistical significance was evaluated using Student’s t-test at *p* ≤ 0.05 in Microsoft Excel (MS Office Professional Plus 2019) and one-way ANOVA was performed under same treatment condition using GraphPad Prism version 10.0.0 (San Diego, CA, USA). Significantly different means were further separated using Duncan’s multiple range test (DMRT) in SAS version 9.4 (SAS Institute Inc., Cary, NC, USA). Statistical comparisons among genotypes were conducted separately within control and heat stress conditions. No direct end-to-end comparisons were made between control samples of one genotype and treated samples of another genotype. Different letters indicate statistically significant differences among genotypes within the same treatment (*p* ≤ 0.05). A heatmap was constructed using TB tools version 2.330.

## 3. Results

### 3.1. Effect of Heat Stress on Relative Water Content, Total Chlorophyll, and Electrolyte Leakage in AtNIA1 and AtNIA2

**Figure 1:**
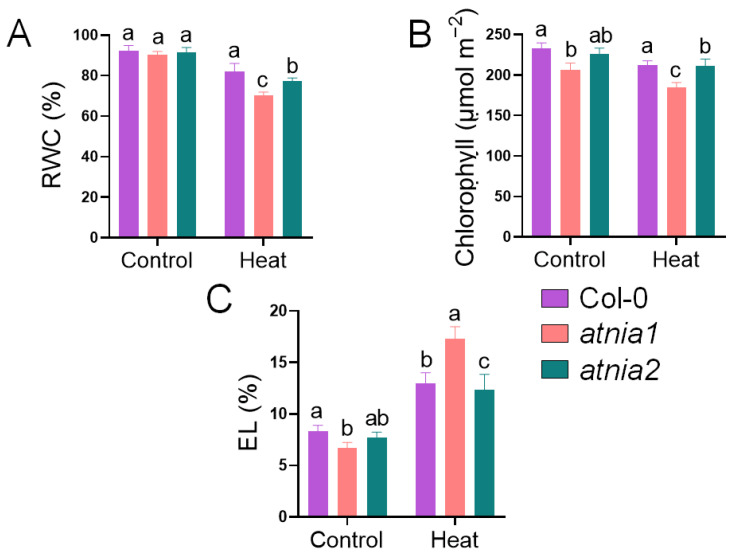
Impact of Heat Stress on (**A**) Relative Water Content, (**B**) Total Chlorophyll, and (**C**) Electrolyte Leakage in *atnia1* and *atnia2*. Data represent the mean of three replicates. Bars with different letters indicate significant differences according to DMRT at *p* ≤ 0.05.

Loss of *AtNIA1* or *AtNIA2* resulted in a significant decrease in relative water content and chlorophyll levels compared to Col-0 under heat stress, indicating impaired stress tolerance in both mutants. Notably, *atnia1* knockout mutant exhibited a more pronounced reduction in these parameters, suggesting heightened sensitivity to heat stress (Figure 1A,B). Regarding electrolyte leakage (EL), no significant difference was observed between Col-0 and *atnia2* knockout mutant, whereas *atnia1* knockout mutant showed markedly elevated EL (Figure 1C). These results suggest that a functional *AtNIA1* gene may play a critical role in maintaining cellular integrity under heat stress conditions.

### 3.2. Phenotypic Response of AtNIA1 and AtNIA2 Under Heat Stress

**Figure 2:**
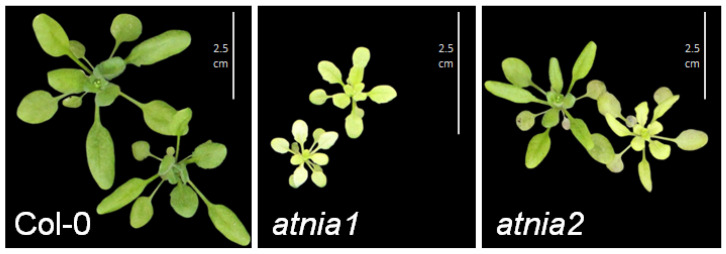
Visual phenotypic differences in plant size and leaf coloration under heat stress for 5 days.

Upon exposure to heat stress, wild-type Col-0 plants maintained robust growth and exhibited greener leaves, whereas the *atnia1* and *atnia2* knockout mutants displayed noticeable leaf yellowing (Figure 2). The smaller stature observed in the *atnia1* mutant may reflect disruptions in nitrogen metabolism and growth processes, suggesting that both *AtNIA1* and *AtNIA2* contribute positively to heat stress tolerance. The chlorophyll content presented in Figure 1 provides objective support for the stress-induced yellowing effects observed in the mutants.

### 3.3. AtNIA1 and AtNIA2 Show Differential Response Under Heat Stress on Oxidative Damage and Antioxidant Enzyme Activities

**Figure 3:**
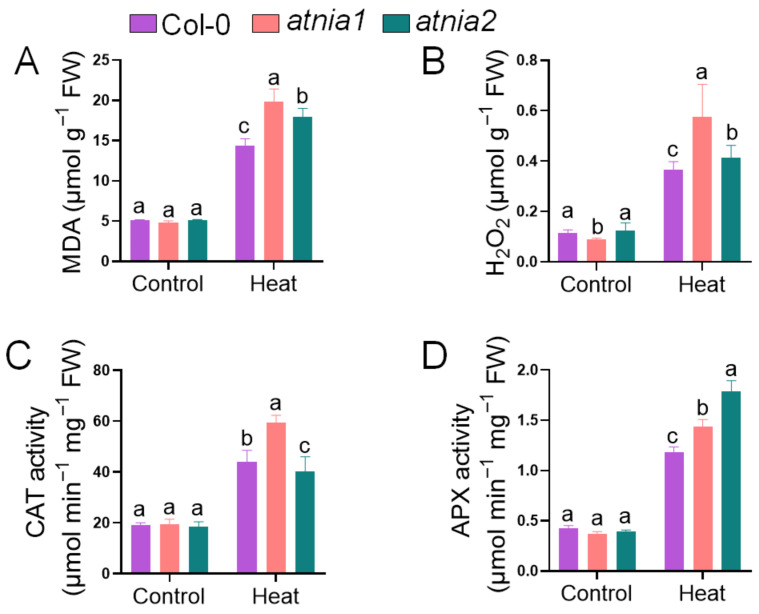
Impact of Heat Stress on (**A**) malondialdehyde (MDA), (**B**) hydrogen peroxide (H_2_O_2_) (**C**) catalase (CAT) activity, and (**D**) ascorbate peroxidase (APX) activity in *atnia1* and *atnia2*. Data represent the mean of three replicates. Bars with different letters indicate significant differences according to DMRT at *p* ≤ 0.05.

To determine whether the observed physiological impairment was associated with oxidative stress, lipid peroxidation and hydrogen peroxide levels were analyzed. In response to heat stress, *atnia1* knockout plants exhibited the highest levels of (A) malondialdehyde content, (B) hydrogen peroxide (H_2_O_2_), followed by *atnia2* knockout mutant, compared to wild-type Col-0. Heat stress resulted in increased accumulation of MDA and H_2_O_2_ in *atnia1* and *atnia2* knockout mutants compared with Col-0, indicating enhanced oxidative damage in nitrate reductase-deficient plants (Figure 3A,B). Catalase (CAT) activity was boosted in *atnia1* knockout mutants, intermediate in Col-0, and lowest in *atnia2* knockout mutants, suggesting differential regulation of CAT under heat stress. Conversely, ascorbate peroxidase (APX) activity was elevated in *atnia2* knockout plants, followed by *atnia1* mutant, highlighting distinct modulation of antioxidant enzymes in response to elevated temperatures.

### 3.4. Transcript Accumulation of Heat Shock Related Proteins and Multiprotein Bridging Factor Gene

**Figure 4:**
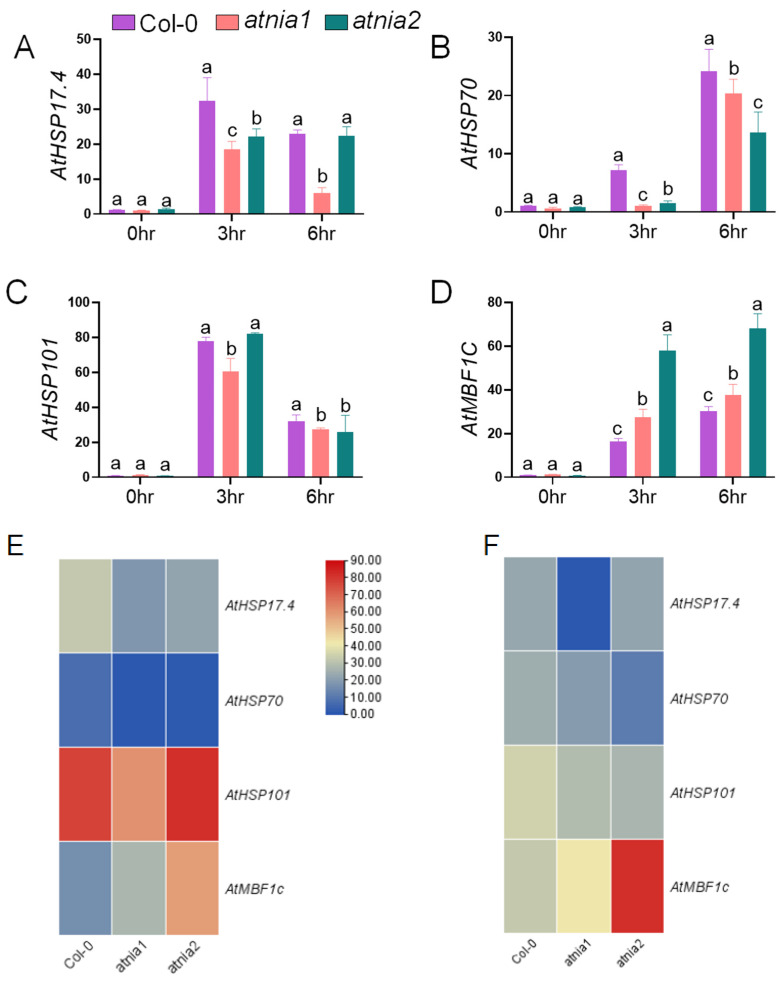
Relative expression of heat stress-responsive genes in *atnia1*, *atnia2*, and Col-0. (**A**) *AtHSP17.4*, (**B**) *At*HSP70, (**C**) *AtHSP101*, and (**D**) AtMBF1c. Data represent the mean of three replicates. Bars with different letters indicate significant differences according to DMRT at *p* ≤ 0.05. Heatmap showing relative expression of heat-responsive genes following heat treatments: (**E**) 3 h and (**F**) 6 h exposure at 37 °C. The heatmap was generated using TBtools based on mean expression values from three biological replicates. Red and blue colors indicate high and low transcript levels, respectively, representing the intensity of gene expression in response to treatments. Genes analyzed include *AtHSP17.4*, *AtHSP70*, *AtHSP101*, and *AtMBF1c* across the genotypes Col-0, *atnia1*, and *atnia2*.

Next, the transcript levels of representative heat-responsive genes (*HSP17.4*, *HSP70*, *HSP101*, and *MBF1c*) were analyzed after 3 h and 6 h of heat exposure (Figure 4A–D). After 3 h of heat treatment, both *atnia1* and *atnia2* knockout mutants exhibited significantly lower expression of *HSP17.4* and *HSP101* compared with their levels at 6 h, indicating time-dependent induction. In contrast, *HSP70* expression was markedly upregulated in both mutants at 6 h. These temporal expression patterns suggest differential regulation of early and late heat stress responses, particularly in nitrate reductase-deficient plants.

Notably, *MBF1c* transcript levels were significantly higher in both mutants than in wild-type Col-0, with more pronounced induction at 6 h, highlighting its potential involvement in NR-mediated heat stress tolerance. The strong late-phase induction of *MBF1c*, especially in *atnia2*, suggests activation of a distinct transcriptional regulatory program during prolonged heat stress.

The heat map further illustrates clear time- and genotype-dependent expression patterns. At 3 h (Figure 4E), *HSP101* showed strong induction, whereas *HSP70* remained relatively low (numerical data presented in Table S2). At 6 h (Figure 4F), expression profiles shifted, with sustained or enhanced *MBF1c* induction in *atnia2*, supporting differential temporal regulation of heat stress responses among genotypes.

### 3.5. Heat Stress Induced Changes in ABA and SNO Mediated by AtNIA1 and AtNIA2

**Figure 5:**
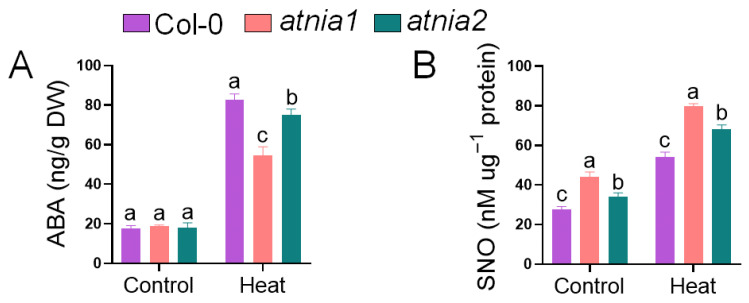
Impact of Heat Stress on (**A**) Abscisic acid (ABA) content, and (**B**) S-nitrosoglutathione content in *atnia1* and *atnia2*. Data represent the mean of three replicates. Bars with different letters indicate significant differences according to DMRT at *p* ≤ 0.05.

Under heat stress conditions, abscisic acid (ABA) content differed significantly among the genotypes. The *atnia2* knockout mutant exhibited the highest ABA accumulation, followed by Col-0, whereas the *atnia1* knockout mutant showed the lowest ABA level (Figure 5). These results indicate a genotype-dependent variation in ABA response to heat stress, with *atnia2* displaying a more pronounced induction compared to the wild-type. In contrast, S-nitrosothiol (SNO) content showed an opposite trend. Under both control and heat stress conditions, SNO levels were highest in the *atnia1* knockout mutant, followed by *atnia2*, and were lowest in Col-0. Heat stress further elevated SNO accumulation across all genotypes. This pattern suggests that disruption of *NIA* genes promotes enhanced SNO accumulation irrespective of temperature conditions, with a stronger effect observed in *atnia1* knockout mutant.

## 4. Discussion

Plants are constantly exposed to fluctuating environmental conditions, and among these, heat stress has emerged as a major constraint on crop productivity worldwide [29]. Our study investigated the differential roles of *AtNIA1* and *AtNIA2* in thermotolerance by comparing heat-induced physiological and molecular changes in *Arabidopsis thaliana* wild-type (Col-0) and nitrate reductase knockout mutants (*atnia1* and *atnia2*). The results revealed that *atnia1* mutants displayed greater sensitivity to heat stress than *atnia2*, suggesting that *AtNIA1* plays a more pivotal role in mediating heat stress tolerance.

High-temperature exposure is known to cause oxidative damage by increasing the production of reactive oxygen species (ROS), which are cytotoxic at elevated levels [30]. Malondialdehyde (MDA) is widely recognized as a reliable indicator of ROS-induced lipid peroxidation and membrane damage. In the present study, both *atnia1* and *atnia2* knockout mutants exhibited elevated MDA accumulation under heat stress, with a significantly greater increase observed in *atnia1*. This finding aligns with previous reports showing that *atnia1* and *atnia2* knockout mutants accumulate excessive ROS under salinity, oxidative stress, and cold acclimation conditions, resulting in impaired membrane integrity and enhanced stress sensitivity [10,11,13]. The increased MDA content in *atnia1* indicates a severe disruption in redox homeostasis, likely due to impaired nitric oxide (NO) production. Nitric oxide plays a central role in alleviating oxidative stress through its ability to directly scavenge ROS and modulate the activity of antioxidant enzymes [8]. *AtNIA1*, in particular, has been implicated as a major contributor to NO biosynthesis under stress conditions, whereas *AtNIA2* is primarily involved in nitrate assimilation under normal physiological states [12]. The more pronounced heat-sensitive phenotype observed in atnia1 mutants in this study further supports the distinct functional role of NIA1 in stress adaptation. Reduced NO availability in atnia1 likely compromises the activation of protective antioxidant defenses, thereby exacerbating oxidative stress and resulting in increased cellular damage under heat conditions.

In addition to oxidative damage, heat stress severely impacts chlorophyll content, affecting photosynthetic efficiency. Our results show a marked decline in chlorophyll levels in *atnia1* mutants compared to *atnia2* and Col-0 under heat stress. This decline may result from increased photo-oxidative damage in chloroplasts due to impaired ROS scavenging and insufficient protection of photosynthetic machinery. The maintenance of higher chlorophyll content in *atnia2* suggests partial retention of NO-mediated protective mechanisms, further highlighting the dominant role of *AtNIA1*. ABA-triggered hydrogen peroxide production stimulates the expression of *NIA1* and *NIA2*, promoting nitric oxide (NO) synthesis, which is essential for ABA-mediated stomatal closure [12]. NO further modulates ABA signaling by inhibiting the PYR/PYL receptors, thereby fine-tuning the ABA response under various stress conditions [31]. Arabidopsis plants lacking NO, such as the *atnia1 nia2 noa1-2* triple mutant, display heightened sensitivity to ABA during both development and stress conditions.

At the molecular level, expression analysis of heat-responsive and antioxidant-related genes revealed downregulation or delayed induction in *atnia1* compared to Col-0 and *atnia2*. Genes such as *HSP70*, *HSP101*, and *HSP17.4* are known to be upregulated under heat stress and are regulated by redox signals including NO and H_2_O_2_ [32,33,34,35]. The suppressed transcriptional levels of these genes in *atnia1* under heat stress may explain the heightened oxidative damage and reduced thermotolerance observed in this genotype. Overexpression of *AtNIA2* in a *Cyclic Nucleotide-Gated Ion Channel 6* (CNGC6) mutant background led to enhanced expression of heat shock proteins *HSP17.7* and *HSP21*, suggesting a positive role for *AtNIA2* in thermotolerance through stress-responsive gene regulation [36]. In contrast, the *atnia1nia2* double mutant exhibited heightened sensitivity to thermostress, accompanied by a significant reduction in *HSP18.2* transcript levels, further highlighting the importance of nitrate reductases in the heat stress signaling network [37]. Consistent with previous findings that MBF1c acts as a key regulator of thermotolerance in *Arabidopsis thaliana*, the observed upregulation of MBF1c under heat stress suggests its crucial role in activating protective responses [38,39]. The results demonstrate that disruption of nitrate reductase activity compromises heat stress tolerance, with *atnia1* showing greater sensitivity than *atnia2*. The pronounced late-phase induction of MBF1c in *atnia2* suggests the activation of a compensatory transcriptional program during prolonged heat stress. Such late-stage responses are distinct from early heat shock reactions and likely reflect adaptive reprogramming rather than immediate protection. Given that ABA is required for the accumulation of MBF1c and APX1 during combined water deficit and heat stress, this indicates that MBF1c-mediated thermotolerance may be closely linked to ABA-dependent signaling pathways [39]. The pronounced upregulation of MBF1c in *atnia2* and, to a lesser extent, in *atnia1* knockout lines under heat stress may indicate a compensatory activation of thermotolerance pathways in the absence of functional nitrate reductase activity. Since MBF1c is ABA-dependent under combined abiotic stresses, this response could reflect a shift toward ABA-mediated protective signaling when NO production via *NIA1* and *NIA2* is impaired. Consistent with this interpretation, the altered ABA and SNO accumulation patterns observed in *atnia1* and *atnia2* knockout mutants suggest that disruption of nitrate reductase isoforms affects the coordination between hormonal and nitric oxide-related pathways during heat stress. The elevated SNO levels in the mutants, particularly in atnia1 knockout mutant, point to enhanced nitrosative signaling, which may become detrimental if not tightly regulated. Interestingly, the increased SNO accumulation in atnia1 despite its reduced nitrate reductase-dependent NO production may reflect the contribution of alternative NO-generating pathways or altered SNO turnover under heat stress conditions. Heat stress may also influence the balance between SNO formation and degradation, potentially leading to enhanced accumulation of S-nitrosothiols even when NR-derived NO production is compromised. At the same time, genotype-specific differences in ABA accumulation imply distinct regulatory contributions of NIA1 and NIA2 to stress-responsive hormonal signaling. Collectively, these physiological and transcriptional changes support the notion that precise coordination between ABA signaling and NO/SNO homeostasis is critical for thermotolerance. Disruption of nitrate reductase-dependent pathways appear to influence both the timing and magnitude of stress-responsive gene expression, and imbalance in this regulatory network may shift the plant toward a more stress-sensitive phenotype.

**Figure 6:**
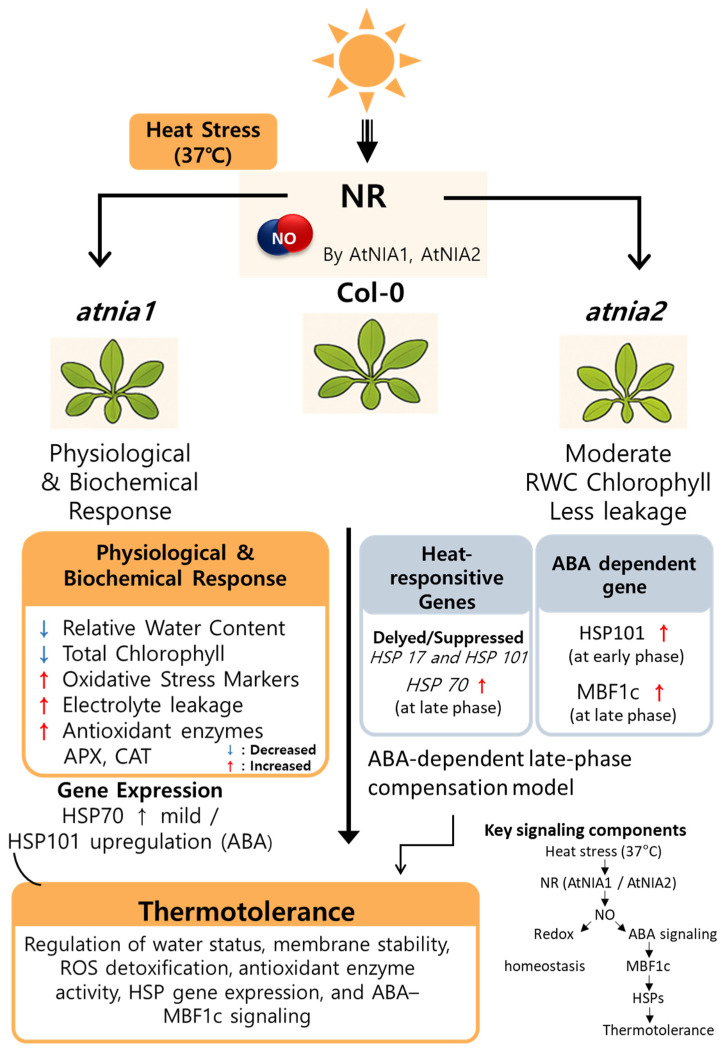
Proposed model illustrating the role of nitrate reductase-dependent nitric oxide signaling in *Arabidopsis* heat stress tolerance. Heat stress induces nitrate reductase (NR) activity mediated by *AtNIA1* and *AtNIA2*, leading to nitric oxide (NO) production. NR-derived NO contributes to the regulation of physiological and biochemical responses, including redox homeostasis and chlorophyll stability, as well as the expression of heat-responsive and ABA-dependent genes. In the absence of functional NR, plants exhibit altered stress-responsive gene expression and reduced thermotolerance.

Furthermore, our results suggest that NIA1 and NIA2 may contribute differently to stress regulation. The stronger heat sensitivity and altered NO/SNO balance observed in *atnia1* knockout mutant imply a more prominent role for NIA1 in early NO-mediated stress signaling. In contrast, the delayed MBF1c induction in atnia2 knockout mutant suggests that NIA2 may participate in modulating the timing or amplitude of transcriptional responses under prolonged stress (Figure 6). Thus, the two NR isoforms appear to have partially overlapping but functionally distinct roles, potentially acting at different phases of the heat stress response or influencing separate regulatory branches within the ABA–NO signaling network.

## 5. Conclusions

Collectively, our findings demonstrate that *AtNIA1* plays a critical role in heat stress tolerance by contributing to NO-mediated antioxidant defense, chlorophyll preservation, and stress gene regulation. Although *AtNIA2* also contributes to stress responses, its effect appears to be less prominent under heat stress conditions. This functional divergence highlights the importance of isoform-specific studies in stress physiology and opens new avenues for improving heat resilience in plants through targeted manipulation of nitrate reductase pathways.

## Data Availability

The original contributions presented in this study are included in the article/Supplementary Material. Further inquiries can be directed to the corresponding author.

## Supplementary Materials

The following supporting information can be downloaded at https://www.mdpi.com/article/10.3390/biom16030415/s1, Table S1. Primer list used in the experiment for quantitative real time PCR. Table S2. Numerical mean values of transcript levels presented in heatmap.
