# Supplementary material for: Nitrate Reductase Genes AtNIA1 and AtNIA2 Confer Heat Stress Resilience via ROS Homeostasis and HSP Expression in Arabidopsis
Source: Biomolecules. 2026 Mar 11;16(3):415. doi: 10.3390/biom16030415 (PMC13023476; doi:10.3390/biom16030415)
Supplement: Supplementary file 1 [file biomolecules-16-00415-s001.zip › biomolecules-4116159-supplementary.pdf]

Table S1. Primer list used in the experiment for quantitative real time PCR.

Common Name	Forward primer	Reverse primer	Gene no	Description
AtHSP17.4	TCTCCCGATTTCTCGTTCCG	AGTCCTCTCTTCCTCCACTGT	AT1G54050	Heat shock related protein
AtHSP70	GCTGGAAACACCCGAGTTTG	TGAGTCAAGCCGCTTCTCAA	AT4G16660	Heat shock related protein
AtHSP101	GCTGGCAAAACTGAAGGGTC	CCTCCTCTCAAAGGCAGCAT	AT1G74310	Heat shock related protein
AtMBF1c	AAGCGGATTTGGCGAAACAG	TCTCCATCTTCGCAAGCACA	AT3G24500	multiprotein bridging factor 1C
AtPP2A	TATCGGATGACGATTCTTCGTGCAG	GCTTGGTCGACTATCGGAATGAGAG	AT1G69960	Reference gene

Table S2. Numerical mean values of transcript levels presented in heatmap.

	Gene	Col-0	*atnia1*	*atnia2*
3 hour	*AtHSP17.4*	32.33988	18.47631	22.14278
	AtHSP70	7.1306	1.093353	1.480974
	AtHSP101	77.76331	60.64492	81.96681
	AtMBF1c	16.34083	27.42123	57.89159
6 hour	*AtHSP17.4*	23.00383	6.007074	22.30503
	AtHSP70	24.22486	20.36328	13.64568
	AtHSP101	32.09809	27.39947	25.92673
	AtMBF1c	30.18524	37.76166	68.19115
